# The Role and Application of Sirtuins and mTOR Signaling in the Control of Ovarian Functions

**DOI:** 10.3390/cells5040042

**Published:** 2016-11-24

**Authors:** Alexander V. Sirotkin

**Affiliations:** 1 Department of Zoology and Anthropology, Constantine the Philosopher University, 94974 Nitra, Slovakia; asirotkin@ukf.sk; Tel.: +421-90-356-1120; 2 Research Institute of Animal Production, 941 51 Lužianky, Slovakia

**Keywords:** sirtuin, mTOR, ovary, proliferation, apoptosis, hormones, folliculogenesis, cancer

## Abstract

The present short review demonstrates the involvement of sirtuins (SIRTs) in the control of ovarian functions at various regulatory levels. External and endocrine factors can affect female reproduction via SIRTs-mammalian target of rapamycin (mTOR) system, which, via hormones and growth factors, can in turn regulate basic ovarian functions (proliferation, apoptosis, secretory activity of ovarian cells, their response to upstream hormonal regulators, ovarian folliculo- and oogenesis, and fecundity). SIRTs and SIRTs-related signaling molecules and drugs regulating mTOR can be used for characterization, prediction, and regulation of ovarian functions, as well as for diagnostics and treatment of ovarian disorders.

## 1. Introduction

Reproduction is the most important biological process, and enables the existence of species. The main female reproductive organ is the ovary, which produces ovarian follicles. These ovarian follicles undergo development and selection, during which the majority of ovarian follicles are lost due to atresia/apoptosis of ovarian cells, while few follicles complete development, ovulate, and expel mature oocyte/egg. The exhausting of the ovarian reserve (the number of primordial follicles) and the subsequent decrease in the number of growing follicles and quality of their oocytes due to aging, chemotherapy, or other factors results in infertility. These processes are regulated at various regulatory levels by numerous and interrelated endocrine and intracellular signaling molecules [1], including sirtuins (SIRTs) [2].

SIRTs are seven peptides (SIRTs 1–7), members of the NAD+-dependent deacetylase and ADP-ribosyltransferase family whose are present in each organ [3,4,5]. SIRTs control acetylation and ribosylation, and therefore the post-translational modification and biological activity of various regulatory proteins. It is proposed that each member of the SIRT family has its own particular biological role, due to their localization and target molecules within the cell. Deacetylases SIRTs 1 and 2 are located both in cytoplasm and nucleus; SIRTs 3–5, possessing both deacetylase and ADP-ribosyltransferase activity are identified only in mitochondria; and deacetylase and ADP-ribosyltransferase SIRT6 and deacetylase SIRT7 are located in nuclei [5].

SIRTs are able to regulate a wide variety of physiological processes, including metabolism, cell cycle, cell differentiation, apoptosis, and aging [6,7]. SIRTs affect these processes via the down-regulation of a serine/threonine kinase called mammalian target of rapamycin (mTOR) [8,9] and other intracellular signaling pathways [5], although mTOR can in turn up-regulate SIRTs production [9,10,11,12]. Recently, SIRTs and their regulators have been intensively studied to examine the potential of SIRTs to be diagnostic and predictive indexes for some disorders, and to be useful for the treatment of malignant and metabolic disorders. The growing body of evidence suggests that SIRTs could be involved in the control of female reproductive processes, and that SIRTs or their analogues could be applicable for the characterization and regulation of animal and human female reproductive processes and the treatment of reproductive disorders. The studies of SIRTs’ role and application are currently using three main approaches—(1) detection of the association between the expression of SIRTs with ovarian physiological and pathological state; (2) examination of the consequences of down- and up-regulation of SIRTs by pharmacological regulators, cDNA, or siRNA constructs; and (3) application of synthetic or plant mTOR inhibitors, which could promote the production of SIRTs. In this review, we attempted to present a short overview of the current knowledge concerning the involvement of SIRTs in the control of ovarian functions and the treatment of their disorders obtained by using these approaches.

## 2. The Use of SIRTs as Markers of Ovarian Cell State

SIRT1 is present in whole ovarian follicle, ovarian epithelium, and stroma [13] and luteinized granulosa cells [10], while SIRT3 [14] and SIRT5 [15] have been detected in ovarian granulosa cells and cumulus oophorus surrounding the oocyte. The amount of SIRTs in ovarian cells is associated with their state and health. Atresia of porcine ovarian follicles was associated with a decrease in SIRT1 expression [13]. Zhang et al. [2] reported that the expression levels of SIRT1, SIRT3, and SIRT6 were decreased in the ovaries of aged mice and mice treated with chemotherapy, but increased in calorie-restricted mice. Moreover, SIRT1, SIRT3, and SIRT6 expression showed a significantly positive correlation with the numbers of primordial ovarian follicles. The age-dependent reduction in mice ovarian reserve was associated with decreased SIRT1, SIRT3, and SIRT6 expression [2], while the age-dependent decline in women’s ovarian reserve was associated with reduced expression of SIRT3 [14] and SIRT5 [15]. These observations indicate that SIRT1, SIRT3, SIRT5, and SIRT6 can be markers of ovarian reserve and ovarian aging. Calorie restriction, which suppresses murine ovarian follicle development, was associated with a reduction in the follicular content of SIRT1 and SIRT6 [16]. Therefore, SIRTs 1, 3, 5, and 6 are potentially useful markers to characterize and to predict ovarian follicular development and related fecundity. Nevertheless, the expression of these SIRTs was studied in relation to the inhibition of ovarian folliculogenesis induced by food restriction and aging, while the association of these and other SIRTs with reproduction rate in normal conditions seems to not have been examined yet.Such studies would be potentially applicable for early selection of farm animals for high reproductive efficiency.

SIRTs can be markers of the pathological state of ovarian cells. For example, polycystic ovarian syndrome is associated with decreased expression of SIRT1 [17]. High expression of SIRT1 is associated with malignant transformation of human ovarian tissue [18] and with poor outcomes in patients with ovarian carcinoma [19]. Despite the potential usefulness of SIRTs for diagnostics of ovarian dysfunctions, SIRTs other than SIRT1 do not seem to have been studied from this viewpoint.

## 3. The Use of SIRTs to Study, Control, and Treat Ovarian Functions

**Figure 1:**
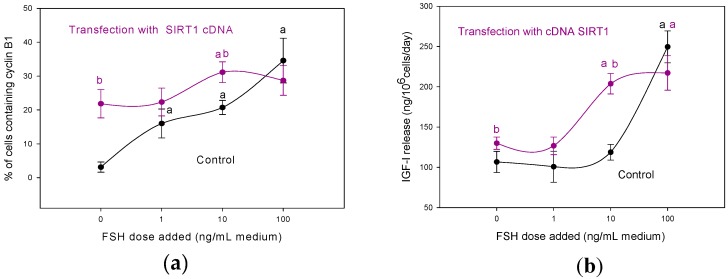
Transfection with sirtuin-1 (SIRT1) cDNA construct promotes proliferation. (**a**) Percentage of cells containing cyclin B1, and (**b**) IGF-I release by porcine ovarian granulosa cells and the response of these cells to follicle stimulating hormone (FSH). A monolayer of granulosa cells of prepubertal gilts was transfected with SIRT1 cDNA or with transfection reagent with empty vector without any gene construct (control). After 30 h of incubation with or without FSH (0, 1, 10, 100 ng/mL medium), the cells and culture medium were analyzed by immunocytochemistry and RIA. Data are the mean ± S.E.M. (**a**) Effect of FSH addition: significant (*p* < 0.05) differences between cells cultured with (1, 10, or 100 ng/mL) and without (0 ng/mL) FSH; (**b**) Effect of transfection: significant (*p* < 0.05) differences between corresponding groups of cells transfected and not transfected with gene construct. Reprinted from *Animal Reproduction Science*, Volume 140, Silvia Pavlová et al., The involvement of SIRT1 and transcription factor NF-κB (p50/p65) in regulation of porcine ovarian cell function, Pages 180–188, Copyright© 2013, with permission from Elsevier.

There is a growing body of evidence showing that the members of the SIRT family can affect ovarian functions, including ovarian cell proliferation, apoptosis, folliculogenesis, oogenesis, and embryo development at various regulatory levels (see reviews [20,21,22]). SIRT1-deficient mice had reduced level of gonadotropin releasing hormone (GnRH) in hypothalamus, luteinizing hormone (LH), and follicle stimulating hormone (FSH) in plasma [22]. Resveratrol-induced SIRT1 expression was associated with increased levels of LH (but not FSH) receptors and steroidogenic enzymes in rat ovaries [10]. These observations suggest that SIRT1 can promote ovarian functions via activation of the GnRH–gonadotropin–ovarian gonadotropin receptor axis, although direct action of SIRT1 on the ovarian cells is also evident. Transfection with a SIRT1 gene construct stimulated proliferation (but not apoptosis), progesterone, testosterone, and IGF-1 release by cultured porcine granulosa cells [23,24] and developmental capacity of mouse and human oocytes [25]. Either FSH or OT additions increased the SIRT1 accumulation in cultured porcine ovarian granulosa cells, while IGF-I addition decreased it. mTOR blockers/stimulators of SIRT1 (see below) were able to modify FSH’s effects on proliferation, apoptosis, and steroidogenesis in these cells. These observations suggest that SIRT1 can modify and mediate the effect of hormonal stimulators on ovarian functions [26]. The ability of SIRT1 to promote ovarian cell proliferation and IGF-I release, as well as to promote FSH action on these processes (from [23]) is illustrated by Figure 1. Fu et al. [27] reported the involvement of another SIRT—SIRT3—in the up-regulation of genes involved in human ovarian folliculo-, luteo-, and steroidogenesis: knockdown of SIRT3 resulted in decreased expression of aromatase, 17β-hydroxysteroid dehydrogenase 1, steroidogenic acute regulatory protein, cholesterol side-chain cleavage enzyme, and 3β-hydroxysteroid dehydrogenase in granulosa cells, and decreased their progesterone release. Aside from mTOR, SIRTs can affect ovarian cell functions via changes in the production of transcription factors p53, NFkB [18,23,25], FOXL2 [28], Noch 3 [29], STAT3, steroid hormone receptors, and other intracellular signaling molecules [5], which in turn affect ovarian proliferation, apoptosis, and steroidogenesis. Moreover, SIRT1 overexpression promoted the effects of transcription factors p53 and NFkB on porcine ovarian cell functions [26]. The mice with knock-down of SIRT1 but not of SIRT3 are infertile [21]. It is proposed that SIRTs can affect ovarian functions and ovarian aging by activating the anti-oxidative processes in the reproductive system—especially in oocytes [21,22]. Therefore, the available data suggest the involvement and potential usefulness of SIRT1 and maybe SIRT3 for the regulation of healthy ovarian functions at the level of hypothalamus, pituitary ovarian gonadotropin receptors, ovarian response to hormones, ovarian cell proliferation, apoptosis, folliculo- and oogenesis, as well as ovarian hormones and transcription factors.

These molecules and processes can be mediators of SIRTs’ action on ovarian aging. Ovarian aging in various species is characterized by a gradual decrease in both the number of follicles and the quality of oocytes. These changes are also associated with a decrease in SIRT1, SIRT3, and SIRT6 in the ovaries of aged mice [2], and in SIRT3 [14] and SIRT5 [15] in aged women.

The pharmacological activation of SIRT1 and SIRT6 prevented the age-dependent exhaustion of rat [11] and mice [12] follicle reserve.

In addition, SIRTs can affect ovarian function due to their antioxidant properties. At least the amount of SIRT3 mRNA in both mice and human oocytes was positively correlated with mitochondrial biogenesis [25], and SIRT3 knockdown markedly elevated reactive reactive oxygen species in human ovarian granulosa cells [27].

The association of SIRTs with ovarian state and their ability to regulate healthy ovarian cell functions suggested the potential usefulness of SIRTs in the treatment of ovarian disorders. For example, the pharmacological up-regulation of SIRT1 suppressed the manifestations of polycystic ovarian syndrome in rats [17]. Inhibition of either SIRT1 [28] or SIRT6 [29] reduced the proliferation of human ovarian granulosa tumor cells. The overexpression of another SIRT—SIRT3—caused suppression, and the knock-down effect promoted the development of metastasis in human ovarian carcinoma cells [30]. Therefore, SIRTs (at least SIRTs 1, 3, and 6) can be potential therapeutic tools for the treatment of the most common ovarian disorders—polycystic ovarian syndrome and cancer.

Taken together, the available data demonstrate that SIRTs can be efficient tools to regulate the functions of healthy ovary via action on hypothalamic GnRH, gonadotropins, gonadotropin receptors, response to gonadotropins, transcription factors, ovarian cell proliferation, apoptosis, and hormone release, as well as via action on ovarian aging and oxidative damage. Furthermore, they can have a large therapeutic potential to treat the most common ovarian dysfunctions. Unfortunately, the large-scale practical application of SIRTs in medicine, assisted reproduction, and animal biotechnology is limited by problems in their delivery or in the direct control of their expression via cDNA and small RNA constructs. The less time- and money-consuming approach is to promote SIRTs’ accumulation via caloric restriction [2,16] or mTOR regulators.

## 4. The Use of mTOR Regulators to Study, Control, and Treat the SIRTs-Dependent Ovarian Functions

SIRTs and SIRTs-dependent processes can be affected not only by direct up- and down-regulation of SIRTs, but also by synthetic or natural (plant-derived) mTOR regulators. The mTOR pathway plays a critical role in the regulation of ovarian cell proliferation, apoptosis, secretory activity, folliculogenesis, and malignant transformation [5,11,26,31,32,33,34,35,36,37,38,39,40,41,42,43,44]. The mTOR and SIRTs are in close mutual functional interrelationships. In non-ovarian cells, the plant mTOR blocker resveratrol can activate SIRTs 1, 3, 4, and 7. Other plant extracts and compounds like quercetin also express both mTOR-inhibiting and SIRT1-activating properties [5]. The mTOR blockers resveratrol and rapamycin can promote the accumulation of both SIRT1 and SIRT6 in ovarian cells [10,11]. On the other hand, SIRT1 can suppress mTOR activity within non-ovarian [8] and ovarian [12] cells. The data concerning interrelationships between mTOR and other SIRTs within the ovary have not yet been reported.

Recent studies demonstrated the action of mTOR inhibitors on various ovarian functions (see [29] for review). Synthetic inhibitors of mTOR suppressed the proliferation of healthy mice granulosa cells [32,33] and healthy porcine granulosa cells [26]. A pharmacological inhibitor of mTOR/activator of SIRTs was able to improve the murine follicle reserve and prolong the ovarian lifespan [12], while its plant analogue rapamycin prolongs the ovarian lifespan in rats [11,20]. Synthetic mTOR blockers were able to inhibit the proliferation of mouse ovarian granulosa cell tumors [32] and human ovarian cancer cells [35,36,37]. These inhibitors were able to promote apoptosis of human ovarian cancer cells [35,37]; however, these data were not confirmed by other studies [36]. Synthetic mTOR inhibitors failed to affect the apoptosis of healthy mouse [33] and porcine [26] granulosa cells and mouse ovarian granulosa cell tumors [34]. These inhibitors were able to suppress porcine granulosa cells’ progesterone and testosterone output [26]. Furthermore, mTOR (like SIRTs) may mediate FSH action on ovarian cells; FSH affected mTOR in rat [38] and porcine [26] granulosa cells, while synthetic mTOR inhibitors prevented the stimulatory influence of FSH and induce the inhibitory effect of FSH on proliferation, apoptosis, and steroidogenesis in porcine granulosa cells [26]. Natural mTOR inhibitor (rapamycin) treatment promoted apoptosis in healthy porcine ovarian cells [13], suppressed follicular growth and maturation in rodents [19,32], and increased the risk of oligomenorrhea in humans [39]. Resveratrol, the other mTOR blocker and activator of SIRTs, was able to induce apoptosis/atresia in porcine ovarian follicles [13]. Rapamycin did not affect the release of progesterone by bovine corpus luteum cells [40], but resveratrol reduced steroidogenesis in rat ovarian theca-interstitial cells [41]. Therefore, the majority of available observations suggest that mTOR blockers can suppress basic functions (proliferation and steroidogenesis), promote apoptosis, and prevent/invert the influence of physiological hormonal stimulator (FSH) on these processes.

The easiest way to stimulate SIRTs, however, is not the application of synthetic or natural mTOR blockers, but simple restriction of caloric intake. It can promote SIRT1 accumulation in various non-ovarian cells [5]. Caloric restriction induces the accumulation of SIRT1 in murine ovaries, which is associated with impairment of ovarian follicle development and follicle loss [16].

These features of mTOR inhibitors can be used for the treatment of ovarian disorders. Liu et al. [42] successfully used resveratrol to delay puberty, save the ovarian follicle reserve, prolong reproductive lifespan, and to prevent age-associated infertility in mice. Both synthetic and natural mTOR inhibitors can be used for the treatment of ovarian cancers [5,35,36,43,45].

Therefore, natural and synthetic mTOR regulators and/or caloric restriction provide a less specific, but time-, work-, and money-consuming alternative to manipulations with SIRT gene constructs to study and affect ovarian functions, as well as to treat premature ovarian insufficiency and cancer. These alternative ways of regulating SIRTs-dependent functions including reproduction remain the most popular and promising ones.

## 5. Conclusions

**Figure 2:**
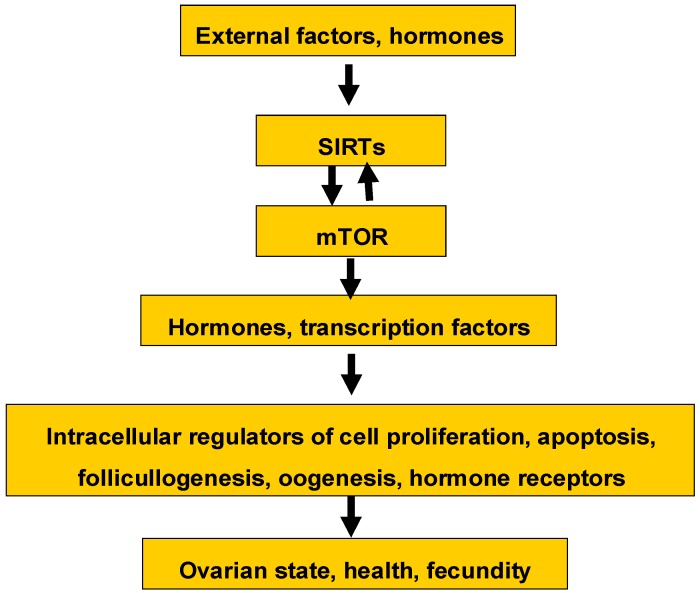
Possible functional interrelationships of SIRTs with intra-ovarian signaling molecules and processes. Explanations are in the text. mTOR: mammalian target of rapamycin.

The present short review of the available knowledge concerning SIRTs in the ovary suggest the involvement of SIRTs in the control of basic ovarian functions at various regulatory levels. The possible interrelationships of SIRTs with other signaling molecules and processes in the control of ovarian functions are shown at Figure 2. These interrelationships, as well as the involvement of SIRTs in the control of all basic ovarian functions, provide a basis for the application of SIRTs and SIRTs-related signaling molecules and drugs for the characterization, prediction, and regulation of ovarian functions, as well as for diagnostics and the treatment of ovarian disorders. Understanding and applying SIRTs is only in the early stages. The majority of the relevant available information concerns only one SIRT—SIRT1—whilst other SIRTs have not been or have been very poorly studied in this respect. Efficient and cheap methods of SIRT analysis and regulation require further development. The most promising and easy way to affect SIRTs and SIRTs-dependent ovarian functions could be the application of synthetic or natural mTOR regulators or the reduction of caloric intake. Targets and mechanisms of SIRTs’ action require further study. Nevertheless, these efforts could be fruitful for the development of a better understanding of the role of SIRTs in the control of reproduction, as well as for their application in biology, biotechnology, and animal and human reproductive medicine.
